# Socioeconomic Differences in the Use of Self-Regulated Learning Strategies: A Population Study

**DOI:** 10.3390/bs15111493

**Published:** 2025-11-03

**Authors:** Giulia Raimondi, Elisa Cavicchiolo, Fabio Alivernini, Fabio Lucidi, Sara Manganelli

**Affiliations:** 1 Department of Developmental and Social Psychology, Sapienza University of Rome, Via dei Marsi 78, 00185 Rome, Italy; giulia.raimondi@uniroma1.it (G.R.); fabio.alivernini@uniroma1.it (F.A.); fabio.lucidi@uniroma1.it (F.L.); sara.manganelli@uniroma1.it (S.M.); 2 Department of Systems Medicine, Tor Vergata University of Rome, Via Montpellier 1, 00133 Rome, Italy

**Keywords:** cognitive strategies, self-regulated learning, measurement invariance, socioeconomic status, latent mean difference

## Abstract

Background: Self-regulated learning (SRL) is a key factor in academic success, with self-regulated cognitive strategies (SRCSs) playing a central role. Identifying the factors linked to low use of SRCSs is therefore essential. Socioeconomic status (SES), a well-established predictor of multiple educational outcomes, may also influence students’ engagement in SRCSs, yet very few studies have explored this issue. Grounded in the SRL framework, this study examines differences in SRCSs use across SES groups. Methods: We analyzed data from the entire population of 10th-grade Italian students (N = 261,255). To ensure that the questionnaire functions equivalently across groups and control for measurement bias and error, Multigroup Confirmatory Factor Analysis was conducted to verify the measurement invariance of the Cognitive Self-Regulation Scale across three SES groups (low, middle, and high), followed by latent mean difference tests. Results: Low-SES students reported markedly lower CSRS use than high-SES peers and also lower use than middle-SES peers. Middle-SES students reported lower use of CSRS than high-SES students. Conclusions: These findings show a clear and consistent impact of SES on the use of SRCSs, potentially contributing to persistent academic disparities, and emphasize the need for interventions to support disadvantaged students, thereby helping to break the cycle of inequality.

## 1. Introduction

In the educational setting, self-regulated learning (SRL) has long been recognized as a central process through which students plan, monitor, and adapt their learning in pursuit of academic goals (Bandura, 2006). Within the SRL framework, learners are viewed as active agents who can regulate cognition, motivation, and behavior (Bandura, 2006), which in turn shape achievement outcomes. In this view, differences in academic success could be explained by the extent to which students employ effective self-regulatory processes during study (Alivernini et al., 2019; Eshel & Kohavi, 2003; Richardson et al., 2012; Schunk, 2005). However, not all students have equal opportunities to develop these regulatory capacities. Socioeconomic disparities may limit access to stimulating learning environments and resources (Alivernini, 2013; Cavicchiolo et al., 2023; Paletta et al., 2017), potentially influencing how students engage in self-regulated learning. Understanding whether and how socioeconomic status relates to the use of self-regulated cognitive strategies is therefore crucial for promoting equity in education.

### Theoretical and Empirical Background

A core component of SRL concerns the regulation of cognition through self-regulated cognitive strategies (SRCSs), which have also long been regarded as a core component of successful academic engagement and lifelong learning (Zimmerman, 2011). These strategies represent concrete, observable behaviors, that play a central role in learning outcomes (Theobald, 2021). Elaboration, networking, and monitoring strategies are generally regarded as forms of deep-level processing (Mega et al., 2014; Soenens et al., 2012) and have long been recognized as central components of self-regulated learning (Pintrich, 2004; Richardson et al., 2012). Elaboration refers to the identification and structuring of key information during study activities (Richardson et al., 2012). Networking involves establishing connections among pieces of information and integrating knowledge from different sources into a coherent structure (Vermunt, 1998). Monitoring, in turn, encompasses metacognitive processes through which students oversee their own learning, evaluating and adjusting cognitive strategies as needed (Pintrich, 2004). These strategies collectively support a deeper and more authentic comprehension of the material to be learned.

Over the past decades, several studies have demonstrated that the use of SRCSs is a key condition for academic success (Manganelli et al., 2019), stronger study motivation (Richardson et al., 2012), greater resilience to academic challenges (Eshel & Kohavi, 2003; Yin et al., 2024), and lifelong learning (Cassidy, 2011). Monitoring and fostering students’ use of SRCSs is therefore essential, particularly among groups that tend to exhibit lower levels of academic achievement and school adjustment.

Students from low socioeconomic backgrounds (SES) typically achieve lower academic results than their higher-SES peers (Berger & Archer, 2016; Broer et al., 2019; Manganelli et al., 2021; OECD, 2014; Vadivel et al., 2023), are less likely to attend higher education (e.g., university degree), are overrepresented in technical/vocational tracks, and more likely to experience grade delay in primary and secondary school (Dietrichson et al., 2017; OECD, 2018; Titus, 2006). SES is a wholesome indicator of the individual’s background, usually measured as a combination of different factors (e.g., the income, educational level, and occupational status of the student’s family). Low-SES students often have limited access to cognitively stimulating environments, fewer educational materials at home, and reduced parental and social support (Brown et al., 2019; Rahal et al., 2023; Vadivel et al., 2023). These disadvantageous conditions are known to compromise students’ educational outcomes, and may also hinder students’ opportunities to develop and internalize effective SRCSs. Low-SES students tend also to report lower academic self-efficacy for SRL (Macphee et al., 2013; Raimondi et al., 2025), which in turn can hinder the use of SRCSs. Collectively, these factors could contribute to generating a recursive cycle in which the lack of SRCSs enhances academic difficulties, further limiting students’ educational opportunities. Conversely, fostering SRCSs among low-SES students could help mitigate the adverse effects of socioeconomic disadvantage on educational outcomes. Indeed, prior research suggest that SES influence both the relationship between motivation and the use of learning strategies (Shin & So, 2018) and the link between self-regulated learning strategies and achievement (Dong, 2023).

Despite the importance of understanding differences in the use of SRCSs across socioeconomic groups, very few studies have addressed this issue, leaving the question open. An analysis of PISA data (OECD, 2024) indicated that socioeconomically advantaged high school students reported greater use of SRCSs compared to their disadvantaged peers. However, these gaps were not uniform across all contexts: in some countries, differences are minimal or even reversed. Furthermore, PISA data pointed to a positive association between the frequency of learning strategies use and higher SES (Callan et al., 2017), with similar associations observed at the middle school level with specific aspects of SES (i.e., parental education and home resources; Akyol et al., 2010). These findings suggest that students from different SES backgrounds may differ in their use of SRCSs; however, the evidence is limited, inconsistent, and subject to certain limitations, thus highlighting the need for further research. For example, the differences identified through PISA analyses (OECD, 2024) compared only the most advantaged and most disadvantaged students, excluding those with middle SES; as a result, whether middle-SES students differ from their low and high-SES peers remains unclear. Furthermore, SRCSs have usually been assessed using single items or composite scale scores derived from summed or averaged item responses, without testing whether the scale or questionnaire demonstrate invariance across groups. Although common in the social sciences, this practice may yield biased results. Establishing measurement invariance is essential to ensure that the questionnaire functions equivalently across groups; otherwise, observed differences may reflect variation in how items are interpreted rather than genuine variation in the underlying construct. Moreover, comparisons based solely on observed scores fail to account for measurement error inherent in questionnaire-based assessments, which can further compromise the reliability of results.

Building on the theoretical framework of SRL, the aim of the current study was to address these limitations by examining differences in SRCSs across students from different SES groups. On the basis of the literature summarized above, we hypothesized that students with higher socioeconomic status would report a greater use of deep-level strategies (e.g., networking and monitoring) compared to those from lower socioeconomic backgrounds.

Several methodological strategies were implemented to respond to gaps in the literature. The data analyzed included the entire population of 10th-grade Italian students (N = 261,255), providing a comprehensive representation of SES levels and reliable estimates of group differences in SRCSs. We considered three groups of students—low, middle, and high SES—to capture the full range of potential difference in SRCSs use. To address our research aim, we first tested the measurement invariance of the Cognitive Self-Regulation Scale (CSRS; Alivernini et al., 2019; Manganelli et al., 2015) across SES groups, a prerequisite for assessing differences across groups. Second, we assessed differences in SRCSs use through latent mean difference analysis. In contrast to earlier findings derived from single items or composite scale scores, this approach ensures more reliable comparisons by accounting for both measurement invariance and measurement error.

## 2. Materials and Methods

### 2.1. Participants and Procedures

The current study analyzed data from the entire population of 261,255 Italian 10th-grade students, who participated in the National Evaluation of learning (INVALSI, 2015). The average age of the students was 15.60 years (SD = 0.76), and 50.1% were females. The distribution of socioeconomic background was approximately normal (Skewness = −0.20; Kurtosis = −0.32).

This national assessment included standardized tests developed by the National Institute for the Evaluation of the Education System (INVALSI), which assessed reading comprehension in Italian and mathematical skills. In addition to these tests, a questionnaire was administered which included the Cognitive Self-Regulation Scale and demographic information. Students completed the questionnaires in class during the first part of a school day. The assessment adhered to the ethical guidelines of INVALSI (INVALSI, 2015), which reviewed and approved the assessment process. Each school obtained informed consent and parental permission according to the assessment protocol of the INVALSI. The data analyzed in the current study are available upon request at https://serviziostatistico.invalsi.it/en/ (accessed on 15 April 2025).

### 2.2. Measures

Self-Regulated Cognitive Strategies. The Cognitive Self-Regulation Scale (CSRS) (Alivernini et al., 2019; Manganelli et al., 2015) (see Table A1 and Table A2 in Appendix A for both original Italian version and the English version of the CSRS) is a self-report questionnaire that measures cognitive self-regulation. A detailed description of the procedures used to construct this scale can be found in Alivernini et al. (2019). The CSRS is composed of three subscales that measure cognitive strategies that have been regarded as core elements of self-regulation of deep-level processing and a fourth subscale “No-strategy” or “control” subscale, which measured the frequency with which students spend their time studying without employing any specific cognitive strategies. This subscale allows us to distinguish between those who do not study at all and those who study using ineffective approaches. Its inclusion improves the accuracy of the measure and provides a useful control for interpreting students’ overall levels of cognitive self-regulation. The three cognitive strategies measured were “Extraction” (identifying relevant information), “Networking” (linking ideas), and “Monitoring” (checking comprehension). Each subscale consists of three items in which students reported how frequently they used each strategy on a 4-point scale ranging from 1 (“Never”) to 4 (“Very often”). CSRS demonstrated strong factorial validity and measurement invariance across gender and immigrant backgrounds, making it a robust tool for investigating group-based differences in the use of cognitive strategies (Alivernini et al., 2019).

Socioeconomic Status (SES). Students’ SES was assessed following the criteria of the (OECD, 2014), which defines four family background indicators. The first indicator is the educational level of parents, and it was measured through students’ self-reports of their parents’ educational attainment and categorized according to the six levels of the International Standard Classification of Education (ISCED) (UNESCO, 2012). The second indicator is the occupational level of parents, and it was derived by coding students’ reports of their parents’ occupations (e.g., manager, teacher, clerk) into six categories ordered by occupational status. The third indicator is home literacy resources, and it was based on students’ reports of the number of books at home, ranging from “0–10 books” to “more than 500 books.” The final indicator is home possessions, and it was based on students’ responses concerning household items and facilities, such as personal computers, internet access, and desks for studying. Final SES scores were computed using factor scores from a principal component analysis of these indices. Students were categorized into low, middle, and high SES groups using tertiles of the SES scores.

### 2.3. Statistical Analyses

In a preliminary phase, a confirmatory factor analysis (CFA) was conducted to test the adequacy of the measurement model of CSRS (Alivernini et al., 2019). The hypothesized model specified a second-order factor structure comprising one second-order factor (cognitive self-regulation) and four first-order factors (Networking, Extraction, Monitoring, and No-Strategy), each measured by three items. A negative second-order loading was anticipated for the No-Strategy subscale. The following indices were used to assess model fit: (1) the Root Mean Square Error of Approximation (RMSEA), with values between 0.05 and 0.08 indicating adequacy of the model, and values above or equal to 0.10 indicating poor fit of the model (Browne & Cudeck, 1992; Hu & Bentler, 1999); (2) the Tucker–Lewis Index (TLI), with values > 0.95 indicating good fit of the model and values of 0.90 and higher an acceptable fit (Bentler & Bonett, 1980); (3) the Comparative Fit Index (CFI), with values > 0.95 indicating good model fit and values of 0.90 and higher an acceptable fit (Bentler, 1990); (4) the Standardized Root Mean Square Residual (SRMR), with values < 0.08 indicating good fit (Yu, 2002); and (5) the Chi-square (χ^2^) test, with *p*-values greater than 0.05 indicating an adequate fit to the data. However, Chi-square is sensitive to sample size, and so *p* values might become significant for large samples (Schumacker & Lomax, 2012).

In the first step of analysis, the measurement invariance of the CSRS across of students’ levels of SES (i.e., low, middle, and high) was examined through a series of hierarchical multigroup CFAs on the second-order factor model (Chen et al., 2005). Firstly, we tested for configural invariance, which assesses whether the same number of factors, defined by the same items, fits the data equally across groups; secondly, we tested for metric invariance, by constraining first- and second-order factor loadings to be equivalent across groups; thirdly, we tested for scalar invariance, by constraining first- and second-order intercepts to be equivalent across groups (Chen et al., 2005; Meredith, 1993). If scalar invariance is obtained, then participants of different groups who have the same value on the latent factor should have equal values on the same items, and differences in the mean of each item are due to differences among participants’ level of the latent factor. Therefore, with scalar invariance, latent mean differences of the latent factor can be compared across groups. To assess the adequacy between the nested models representing different levels of invariance, changes in CFI (ΔCFI < 0.01), RMSEA (ΔRMSEA < 0.015), and SRMR (ΔSRMR < 0.01) were used (Chen, 2007). At each step of the analysis, the adequacy of the nested models was also assessed using the corrected χ^2^ difference test (Satorra & Bentler, 2001).

In the second step of analysis, latent mean differences in SRCSs were compared across groups with different levels of SES. Specifically, for the first round of the analyses, the Low SES category was set as the reference group for mean comparisons with Middle and High SES; subsequently, the reference group was switched to Middle SES for mean comparisons between Middle and High SES. In the analyses, the variances of the groups were constrained to be equal across groups, and standardized mean differences values were looked for so that the results could be interpreted as Cohen’s d.

All the analyses were performed with Mplus 8.3 (Muthén & Muthén, 2017), with the COMPLEX option in order to account for the nested structure of the data. Accordingly, the Maximum Likelihood Robust (MLR) estimator (Satorra & Bentler, 2001) was applied, and differences in χ^2^ were tested using the Satorra–Bentler scaled difference test. The very small amount of missing data (1.5% to 1.8%) was handled with Full Information Maximum Likelihood method in Mplus.

## 3. Results

**Figure A1:**
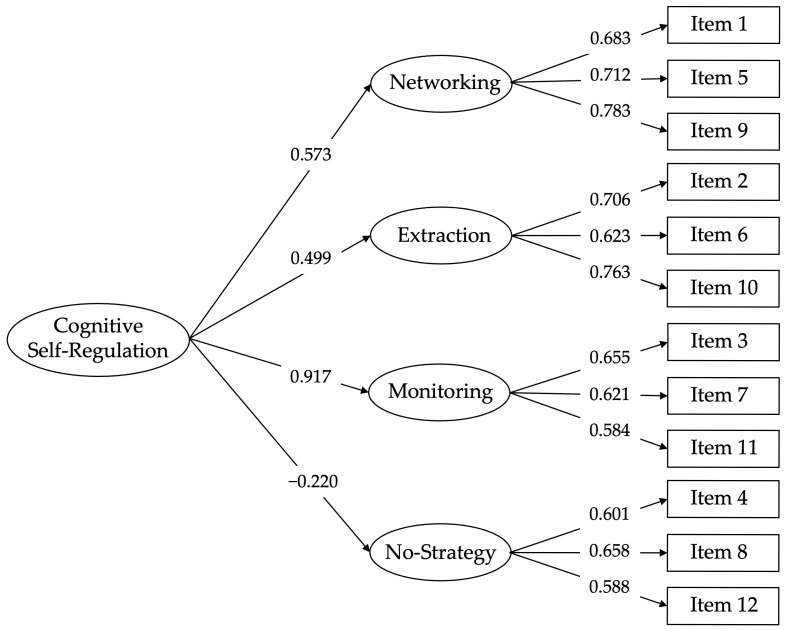
Dimensionality of the CRSR. All the estimates are standardized and statistically significant (*p* < 0.001).

The results of the preliminary CFA (Figure A1 in the Appendix A) confirmed the measurement model of the CSRS. Specifically, the second-order factor model showed a good fit to the data (χ^2^ = 22,454.75, *p* < 0.001; RMSEA = 0.041 [90% confidence interval (CI) = 0.041–0.042]; CFI = 0.96; TLI = 0.95; SRMR = 0.034), with the first- and second-order factor loadings all statistically significant (*p* < 0.001). The internal consistency of the subscales was acceptable (Cronbach’s alpha > 0.60; (Loewenthal, 2004), being 0.77 for Networking, 0.74 for Extraction, 0.65 for Monitoring, and 0.64 for No-Strategy.

The results of the measurement invariance analysis of the CSRS across of students’ SES levels (i.e., low, middle, and high) are reported in Table 1. The fit indices of the unconstrained model (Model 1) demonstrated that the CSRS achieved configural invariance across groups of students with different SES. The results of the analyses on the models with the first-order factor loadings (Model 2) and the second-order factor loadings (Model 4), constrained to be equal across groups, confirmed the full metric invariance of the CSRS across the groups considered. Lastly, the results on the models with the intercepts of the items (Model 3) and intercepts of the first-order factors (Model 5), constrained to be equal across groups, confirmed full scalar invariance across groups of students with different SES.

Since the CSRS demonstrated full invariance, we proceeded to test group difference in latent means on the second-order factor of cognitive self-regulation. As shown in Table 2, students from low-SES backgrounds exhibited significantly lower levels of cognitive self-regulation compared to their peers from both middle SES (*p* < 0.001) and high-SES backgrounds (*p* < 0.001). Moreover, students from middle-SES backgrounds reported using cognitive self-regulation strategies less frequently than those from high-SES backgrounds (*p* < 0.001). Although the effect sizes of these differences are moderate (Cohen, 2013), they represent meaningful disparities at the population level.

## 4. Discussion

The present study aimed to examine the use of self-regulated cognitive strategies (SRCSs) among students from different SES backgrounds. Using the entire population of Italian 10th-grade students, we tested the measurement invariance of the Cognitive Self-Regulation Scale (CSRS) across SES levels to enable rigorous latent mean comparisons. This approach provided interesting results about the association between SES and cognitive self-regulation in high school students.

The results of the first part of our study showed that the CSRS reached scalar invariance across groups of students with different SES backgrounds (i.e., low, middle, and high). This finding is particularly important, as it addresses a methodological gap in the literature: no previous study has ever assessed self-regulation strategies using a measure demonstrated to be invariant across SES groups. Without invariance testing, differences in observed means might reflect biases or artifacts related to the instrument itself (e.g., variations in how items are understood across groups), rather than true differences in the underlying construct (Meredith, 1993; Steinmetz, 2011). Therefore, our results provided empirical support for the SRCSs as a valid and reliable tool for comparing cognitive self-regulation across students from different SES backgrounds. Furthermore, the CSRS made it possible to simultaneously assess three distinct cognitive strategies based on deep-level information processing, yielding a more accurate evaluation. At the same time, the summary score on the second-order factor of cognitive self-regulation facilitates interpretation by clearly highlighting group differences. Finally, the inclusion of the “No-strategy” scale allowed us to obtain a summary score that controls for the frequency with which students dedicate time to studying without relying on effective methods. The CSRS has been confirmed to be a versatile tool that can be applied in multiple settings and for a range of purposes. It may be used for surveying how students learn and can also act as a screening measure for school programs designed to boost academic achievement

In the second part of the study, latent mean comparisons revealed significant differences in the use of self-regulated cognitive strategies across socioeconomic groups. Our findings contributed to the literature by confirming, on the basis of rigorous analytical methods, that low-SES students used cognitive strategies significantly less frequently than their high-SES peers. Moreover, the results showed, for the first time, that the disadvantage in cognitive self-regulation among low-SES students is also evident when compared with middle-SES students. Finally, the findings indicate, again for the first time, that high-SES students exhibited higher levels of cognitive self-regulation even compared to their middle-SES peers. Taken together, these results highlight a clear and consistent impact of SES on cognitive self-regulation, with students from less affluent backgrounds engaging less frequently in effective cognitive strategies than their more advantaged counterparts. Particularly concerning is the magnitude of the difference between low- and high-SES students (Cohen’s d indicates an effect approaching a medium magnitude), as well as the fact that low-SES students also differ significantly from their middle-SES peers, indicating that difficulties in the use of cognitive strategies among disadvantaged students may be especially pronounced.

One possible explanation for this pattern of results is that students from lower SES backgrounds often have limited access to educational resources, including books, technology, and supportive learning environments (Paulus et al., 2021), which can hinder the acquisition and use of SRCSs. In addition, in low-SES families, students may receive less informed academic guidance and may have fewer models of effective learning behaviors or strategies for approaching school tasks (Donnellan et al., 2013). Low SES is also frequently associated with higher levels of stress and instability in the family environment, including financial strain or caregiving responsibilities (Browne & Cudeck, 1992; Rahal et al., 2023). Such stressors can further limit students’ ability to concentrate on schoolwork and employ effective cognitive strategies, ultimately resulting in less frequent use of SRCSs. Finally, students from lower SES backgrounds are more likely to attend schools with poorer facilities, larger class sizes, and fewer extracurricular opportunities (Perry & McConney, 2010), conditions that may discourage them from experimenting with different strategies and developing stronger self-regulatory habits. Taken together, all these challenges reduce students’ opportunities to adopt and use SRCSs, thereby hindering the development of more optimal approaches to learning.

Although individual and family characteristics appear to play a central role, other contextual factors may also help explain the observed SES differences. For example, schools attended mainly by students from less advantaged backgrounds might differ in terms of available resources, learning climate, or the value placed on independent learning, which could influence how students approach study activities. In contrast, students in more privileged schools often benefit from smaller classes, diversified teaching methods, and the use of technologies that promote active engagement and feedback. These aspects could therefore interact with family-related influences, contributing to the overall pattern of results observed in this study.

The reduced use of SRCSs among low-SES students can have serious consequences, including poorer academic performance, reduced resilience to academic challenges, and difficulties in sustaining lifelong learning, all of which may further exacerbate the disadvantaged conditions. Our findings suggest that targeted interventions are needed to foster the use of SRCSs especially among low-SES students, thereby compensating for situations of inequities and supporting those who face unfair disadvantages due to their social background. Unlike SES itself, the use of SRCSs is a malleable factor that can be significantly enhanced through appropriate training programs (Hofer & Shirley, 2003). Recent studies have shown the effectiveness of both narrative-based school programs (Azevedo et al., 2023) and of student tutoring (Vandevelde et al., 2017) in promoting the use of self-regulated learning strategies in students from low socioeconomic backgrounds. Schools and teachers can therefore play a fundamental role in strengthening students’ self-regulation. Creating classroom environments that encourage students to plan, monitor, and evaluate their own learning can help reduce socioeconomic gaps in academic outcomes. Moreover, providing opportunities for guided practice, feedback, and peer learning may enhance the acquisition of SRCSs among students who are less likely to use them spontaneously. By emphasizing these aspects, the study offers evidence that can support the design of school-based initiatives aimed at fostering equitable learning conditions.

Despite its strengths, this study has some limitations that should be acknowledged. First, although it was based on a comprehensive dataset covering the entire population of Italian 10th-grade students, further cross-sectional research is needed to extend the current results to other school grades and cultural contexts. Examining SRCSs use in different educational systems and cultural contexts would help determine whether the patterns observed in the Italian sample are generalizable to students from other countries. Second, because SES also influences academic achievement and other variables related to cognitive self-regulation (e.g., self-efficacy and study motivation) longitudinal studies are needed to clarify the direction and potential reciprocity of these relationships. Thirdly, although SES was operationalized using a composite index, future research could investigate the relative contribution of specific SES components (e.g., parental education vs. income) to the use of SRCSs.

## 5. Conclusions

In conclusion, the present study shows that cognitive self-regulation is significantly associated with SES among high school students, with disadvantaged students reporting less frequent use of self-regulated cognitive strategies. These findings highlight the importance of considering psychological processes in the study of educational inequalities. From an applied perspective, efforts to reduce such inequalities should not be limited to enhancing structural resources (e.g., access to textbooks or technology) but should also explicitly support the development of self-regulatory strategies. The Cognitive Self-Regulation Scale proved to be an effective assessing tool, offering a clear overview of how cognitive strategies are used across different groups and providing teachers with valuable guidance for adapting their approaches to strengthen students’ self-regulated learning skills. Several school-based interventions have demonstrated that structured programs combining cognitive, metacognitive, and motivational components, such as strategy training integrated into classroom activities or the use of reflective learning journals, can effectively enhance students’ self-regulated learning (Cazan, 2022; Dignath & Büttner, 2008). Implementing similar programs may improve their ability to employ self-regulated cognitive strategies effectively, thereby helping to break the cycle of disadvantage.

## Figures and Tables

**Table 1:** Fit indices for the measurement invariance of the CSRS across students’ level of SES (i.e., low, middle, and high).

Model	χ^2^	df	RMSEA	CFI	SRMR	Model Comparison	Δχ^2^	ΔRMSEA	ΔCFI	ΔSRMR
1. Configural	22,211.71	150	0.041	0.960	0.034	-	-	-	-	-
2. First-order factor loadings	22,574.36	166	0.039	0.959	0.034	2 vs. 1	239.15	−0.001	−0.001	0
3. Intercepts of items	23,797.02	182	0.039	0.957	0.035	3 vs. 2	1091.69	0	−0.002	0.001
4. Second-order factor loadings	25,006.28	187	0.039	0.955	0.039	4 vs. 3	1131.10	0	−0.002	0.004
5. Intercepts of first-order factors	25,146.13	193	0.039	0.955	0.039	5 vs. 4	109.94	0	0	0

Note. χ^2^ = Chi-square; df = degree of freedom; RMSEA = Root Mean Square Error of Approximation; CFI = Comparative Fit Index; TLI = Tucker–Lewis Index; SRMR = Standardized Root Mean Residual. All χ^2^ are statistically significant (*p* < 0.001). χ^2^ differences were tested with the Satorra–Bentler scaled difference test. Overall, fit indices indicated that the configural, metric, and scalar models achieved excellent fit, supporting the full measurement invariance of the CSRS across SES groups.

**Table 2:** Results of the Latent Factor Mean Differences tests.

Differences Tests on Cognitive Self-Regulation		
Low SES ^a^ vs. Middle SES	Low SES ^a^ vs. High SES	Middle SES ^b^ vs. High SES
0.168 ***	0.338 ***	0.170 ***

Note. SES = Socioeconomic Status; Low SES = SES first tertile; Middle SES = SES second tertile; High SES = SES third tertile. ^a^ Low SES is the reference group. ^b^ Middle SES is the reference group. *** *p* < 0.001. As shown, latent mean comparisons revealed a clear SES gradient, with low-SES students scoring significantly lower than both middle- and high-SES peers, and middle-SES students scoring lower than those from high-SES backgrounds.

## Data Availability

The data analyzed in the current study are available upon request at https://serviziostatistico.invalsi.it/en/ (accessed on 15 April 2025).

## Appendix A

**Table A1:** English version of the Cognitive Self-Regulation Scale.

	When I Study, I Use My Time …	Never	Sometimes	Often	Very Often
Put a cross in only one box for each question.					
Item 1	to make connections between what I am studying in various different subjects	□_1_	□_2_	□_3_	□_4_
Item 2	to make summaries of the most important things	□_1_	□_2_	□_3_	□_4_
Item 3	to check to see if I have understood an important topic in depth	□_1_	□_2_	□_3_	□_4_
Item 4	to study in general, quickly reading the pages that I have to study without concentrating very much on the concepts	□_1_	□_2_	□_3_	□_4_
Item 5	to look for connections between different concepts	□_1_	□_2_	□_3_	□_4_
Item 6	to write down the most important things	□_1_	□_2_	□_3_	□_4_
Item 7	to check to see if I have properly understood what I’m readings	□_1_	□_2_	□_3_	□_4_
Item 8	to study in general, having a quick look at everything I need to know	□_1_	□_2_	□_3_	□_4_
Item 9	to make connections between the different things that I’m studying	□_1_	□_2_	□_3_	□_4_
Item 10	to make diagrams of the most important things	□_1_	□_2_	□_3_	□_4_
Item 11	to check the parts of a topic that I do not yet know very well	□_1_	□_2_	□_3_	□_4_
Item 12	to study in general, trying to do my homework quickly	□_1_	□_2_	□_3_	□_4_

Note. Networking Subscale: Items 1, 5, 9. Extraction Subscale: Items 2, 6, 10. Monitoring Subscale: Items 3, 7, 11. No-Strategy Subscale: Items 4, 8, 12. (Alivernini et al., 2019).

**Table A2:** Italian version of the Cognitive Self-Regulation Scale.

	Quando Studio, Utilizzo Il Mio Tempo …	Mai	Qualche Volta	Spesso	Molto Spesso
Barra una sola casella per ogni riga.					
Item 1	per collegare le cose che studio in materie diverse	□_1_	□_2_	□_3_	□_4_
Item 2	per fare dei riassunti delle cose più importanti	□_1_	□_2_	□_3_	□_4_
Item 3	per controllare se ho capito fino in fondo un argomento importante	□_1_	□_2_	□_3_	□_4_
Item 4	per studiare in generale, leggendo velocemente le pagine che ci sono da fare senza soffermarmi molto sui concetti	□_1_	□_2_	□_3_	□_4_
Item 5	per cercare dei collegamenti fra concetti diversi	□_1_	□_2_	□_3_	□_4_
Item 6	per annotarmi le cose più importanti	□_1_	□_2_	□_3_	□_4_
Item 7	per controllare se ho capito bene quello che sto leggendo	□_1_	□_2_	□_3_	□_4_
Item 8	per studiare in generale, dando un’occhiata veloce a tutto quello che c’è da sapere	□_1_	□_2_	□_3_	□_4_
Item 9	per fare dei collegamenti tra le diverse cose che sto studiando	□_1_	□_2_	□_3_	□_4_
Item 10	per fare degli schemi delle cose più importanti	□_1_	□_2_	□_3_	□_4_
Item 11	per controllare quali parti di un argomento non so ancora bene	□_1_	□_2_	□_3_	□_4_
Item 12	per studiare in generale, cercando di fare velocemente i compiti	□_1_	□_2_	□_3_	□_4_

Note. Networking Subscale: Items 1, 5, 9. Extraction Subscale: Items 2, 6, 10. Monitoring Subscale: Items 3, 7, 11. No-Strategy Subscale: Items 4, 8, 12. (Alivernini et al., 2019).
